# A case of a patient with erythema annulare centrifugum found to have aggressive fibromatosis

**DOI:** 10.1016/j.jdcr.2024.11.012

**Published:** 2024-11-27

**Authors:** Kathryn Haran, Allison Kranyak, Chandler E. Johnson, Payton L. Smith, Stephanie Hunt, Wilson Liao, Tina Bhutani

**Affiliations:** Department of Dermatology, University of California San Francisco, San Francisco, California

**Keywords:** annular dermatoses, desmoid tumor, healthcare access, paraneoplastic

## Introduction

Erythema annulare centrifugum (EAC) is a type of annular dermatosis commonly associated with malignancy and infections.^1^^,^^2^ Morphologically, it consists of an urticarial papule that undergoes centrifugal spreading and can include a trailing scale.^2^ Due to its similar morphological presentation to other annular dermatoses such as erythema marginatum and annular psoriasis, EAC should be considered in the differential diagnosis of these conditions.^2^ Diagnosis follows a biopsy, with histologic differences between superficial and deep variations.^2^^,^^3^ If superficial, as in this case, biopsy shows a pseudo-vasculitis with perivascular inflammatory infiltrate in a “coat-sleeve” distribution and parakeratosis.^2^ When associated with malignancy, it is known as a paraneoplastic erythema annulare centrifugum eruption (PEACE).^4^ Treatment includes treating the underlying condition and topical steroids.^2^

Aggressive fibromatosis, also known as a desmoid tumor (DT), is a rare type of mesenchymal soft tissue neoplasm.^5^ The incidence of DTs is reported to be 3-5 cases per million people worldwide and has a known association with familial adenomatous polyposis.^5^^,^^6^ Mortality, while uncommon, is often associated with compression and infiltration of surrounding structures.^6^ Patients are diagnosed following pathological evaluation demonstrating long infiltrative fascicles of heterogenous cells and dense collagenous stroma with immunohistochemistry positive for smooth muscle actin and β-catenin.^5^

Here, we present a unique case of a patient diagnosed with EAC preceding a diagnosis of DT. While EAC is a known paraneoplastic syndrome, no studies have reported EAC's presence in conjunction with DT.

## Case

**Fig 1:**
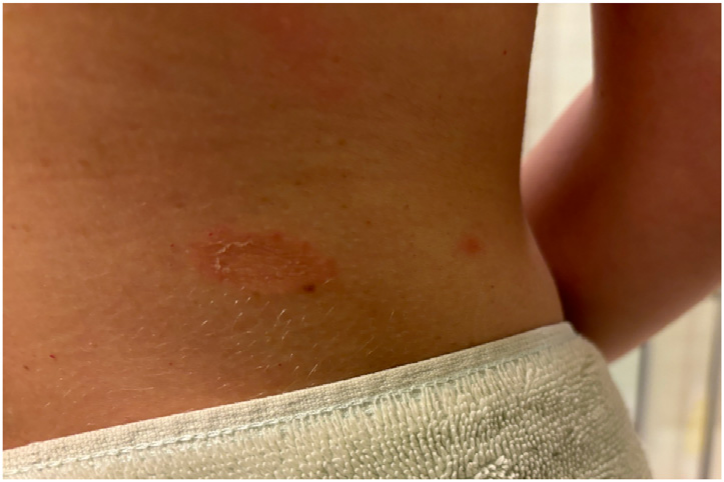
Erythematous annular patch with trailing scale on the *lower* back.

**Fig 2:**
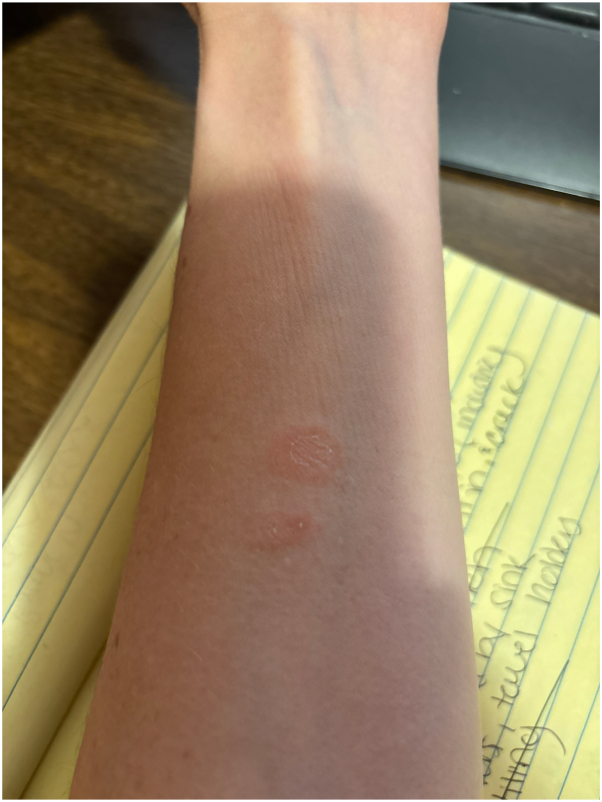
Two annular erythematous plaques with trailing scale on the inner forearm.

**Fig 3:**
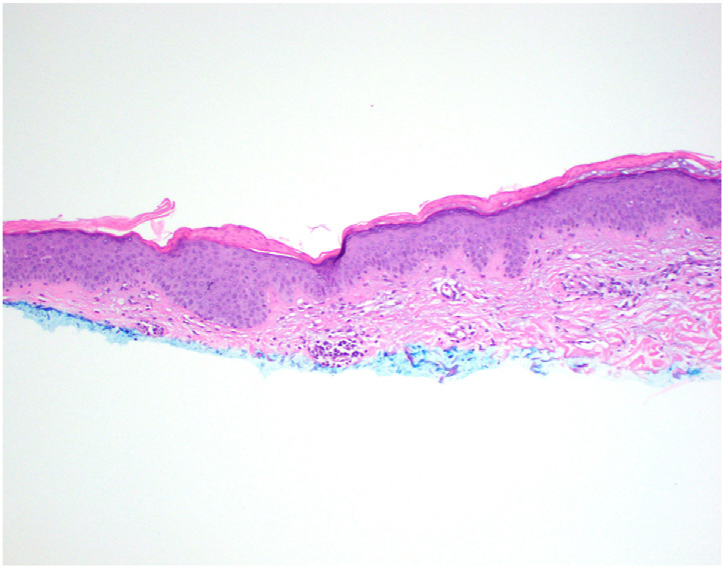
Histopathology of biopsied lesion showing mounded parakeratosis and few perivascular inflammatory cells.

A 35-year-old female with no significant past medical history and no new medications presented to primary care with erythematous, asymptomatic, annular patches on her left shoulder and central back (Fig 1) in October 2023. She was prescribed clotrimazole cream (two times a day) for suspected tinea corporis infection. Following 1 month of treatment, the lesions did not improve and spread down her arms and legs (Fig 2). She was referred to dermatology but could not schedule an appointment until August 2024. In the meantime, she started using clobetasol propionate 0.05% cream (two times a day), which led to fading of the rash. However, new lesions continued to appear over several months. She fortunately received an earlier appointment in April 2024 following a cancellation. A shave biopsy of a lesion on her left thigh revealed slight epidermal spongiosis and basket-to-compact orthokeratosis with foci of mounded parakeratosis consistent with EAC (Fig 3). Only sparse inflammation was noted, however the patient had been using the clobetasol propionate 0.05% cream to the biopsied lesion. Periodic acid-schiff with diastase stain was negative for fungal organisms, and the patient was diagnosed with EAC.

While awaiting her dermatology appointment, in March 2024, the patient also noticed a firm lump on her abdomen, and an ultrasound revealed an irregular hypoechoic lesion with indistinct margins within the rectus musculature that measured 2.0 × 1.2 × 1.6 cm. Following the skin biopsy and diagnosis of EAC, the patient underwent an ultrasound-guided biopsy of the abdominal mass, which showed desmoid-type fibromatosis and tested positive for β-catenin antibodies leading to a diagnosis of a DT. The patient and her care team will determine how to manage her diagnosis in the coming months, be that surgical removal or continued monitoring, and the patient will continue to apply the topical steroids to skin lesions as necessary. An extensive investigation for a separate underlying cause of EAC was negative. This included a negative antinuclear antibody and pregnancy test, a normal chest radiograph, complete blood count, and c-reactive protein levels. A computed tomography scan with contrast revealed no additional lesions apart from the DT.

## Discussion

To our knowledge, this is the first reported case of EAC associated with a DT. This case highlights 2 topics: (1) aggressive fibromatosis should be considered in patients with a soft tissue mass and EAC, and (2) the importance of accessibility to dermatologic care.

In this case, the presence of EAC prior to the identification and diagnosis of the DT suggests a PEACE. Common malignancies in PEACE include lymphoproliferative malignancies such as lymphoma and leukemia but can also less commonly include solid tumors.^2^^,^^4^ It is reported that 28% of abdominal wall DTs will spontaneously resolve without treatment, but the majority of DTs stabilize.^6^ Surgical removal is no longer recommended in the treatment of DTs due to their high recurrence rate, with active surveillance recommended as the first-line treatment approach.^6^ Unfortunately, in this case, since the underlying cause of the EAC is likely the DT, the EAC will likely not resolve as long as the DT is still present.^2^ This demonstrates the contradiction in the best treatment approach for the neoplastic condition compared to the dermatological condition and the importance of interdisciplinary care and shared decision-making with the patient.^2^ It is possible, however, if the DT is treated and the EAC disappears, to confirm the paraneoplastic nature of EAC in this case. Thus, close dermatology follow up is mandatory as this patient manages her DT.

Finally, there is a dichotomy between the demand for dermatologic care and the number of dermatologists, resulting in delayed diagnoses and treatment.^7^ This shortage is particularly impactful in rural areas.^8^ In this case, the patient lived in a rural area and originally had to wait 10 months to be seen for a dermatology appointment. An earlier diagnosis of EAC and systemic work-up may have expedited the diagnosis and discovery of the DT. At baseline, due to their rarity and variable clinical presentation, there is often a delay in the diagnosis of DTs, with patients having to see multiple providers.^6^ This can further impact the quality of life for patients, with DT symptoms including irritability, pain, and anxiety/depression.^6^ In this case, the patient experienced anxiety following the discovery of an unknown abdominal mass, which had a profound impact on her quality of life, including difficulty eating and working.

In conclusion, EAC and DTs are rare entities that can significantly impact quality of life, and this case presents the first incidence of EAC preceding DT. An earlier diagnosis of EAC may have raised clinical suspicion for PEACE, resulting in a systemic workup and the diagnosis of her DT. Continued efforts should focus on increasing rural access to dermatological care.

## Conflicts of interest

Dr Bhutani has received research funding from Amgen, Castle, CorEvitas, Novartis, Pfizer, and Regeneron. She has served as an advisor for Abbvie, Arcutis, Aslan, Boehringer-Ingelheim, Bristol Myers Squibb, Dermavant, Galderma, Incyte, Janssen, Leo, Lilly, Pfizer, Novartis, Sanofi, Sun, Takeda, and UCB. She is a speaker for Janssen. Dr Liao has received research grant funding from Amgen, Janssen, Leo, Novartis, Pfizer, Regeneron, and TRex Bio. Authors Haran, Johnson, Smith, Hunt, and Dr Kranyak have no conflicts of interest to declare.
